# Novel Peripherally Restricted Cannabinoid 1 Receptor Selective Antagonist TXX-522 with Prominent Weight-Loss Efficacy in Diet Induced Obese Mice

**DOI:** 10.3389/fphar.2017.00707

**Published:** 2017-10-05

**Authors:** Wei Chen, Fengchun Shui, Cheng Liu, Xinbo Zhou, Wei Li, Zhibing Zheng, Wei Fu, Lili Wang

**Affiliations:** ^1^Beijing Institute of Pharmacology and Toxicology, Beijing, China; ^2^State Key Laboratory of Toxicology and Medical Countermeasures, Beijing, China; ^3^Department of Medicinal Chemistry, School of Pharmacy, Fudan University, Shanghai, China

**Keywords:** CB1 receptor, antagonist, obesity, periphery, blood–brain barrier

## Abstract

The clinical development of the first generation of globally active cannabinoid 1 receptor (CB1R) antagonists was suspended because of their adverse neuropsychiatric effects. Selective blockade of peripheral CB1Rs has the potential to provide a viable strategy for the treatment of severe obesity while avoiding these central nervous system side effects. In the current study, a novel compound (TXX-522) was rationally designed based on the parent nucleus of a classical CB1R-selective antagonist/inverse agonist, rimonabant (SR141716A). Docking assays indicate that TXX-522 was bound with the CB1R in a mode similar to that of SR141716A. TXX-522 showed good binding, CB1R-selectivity (over the CB2R), and functional antagonist activities in a range of *in vitro* molecular and cellular assays. *In vivo* analysis of the steady state distribution of TXX-522 in the rat brain and blood tissues and the assay of its functional effects on CB1R activity collectively showed that TXX-522 showed minimal brain penetration. Moreover, the *in vivo* pharmacodynamic study further revealed that TXX-522 had good oral bioavailability and a potent anti-obesity effect, and ameliorated insulin resistance in high-fat diet-induced obese mice. No impact on food intake was observed in this model, confirming the limited brain penetration of this compound. Thus, the current study indicates that TXX-522 is a novel and potent peripherally acting selective CB1R antagonist with the potential to control obesity and related metabolic disorders.

## Introduction

Obesity has undoubtedly become a global concern, affecting more than 1.1 billion individuals worldwide (Silvestri and Di Marzo, 2012; Ahmad and Edwards, 2016). However, current pharmacological approaches to morbid obesity are fairly limited. The endocannabinoid system and particularly, the cannabinoid receptors and endocannabinoids, is involved in the regulation of multiple functions including metabolism and energy homeostasis (Janero and Makriyannis, 2009; Kendall and Yudowski, 2016; Knani et al., 2016). In addition to its broad distribution in the central nervous system, cannabinoid receptor 1 (CB1R) is also expressed by numerous peripheral organs and tissues including the liver, adipose tissue, gastrointestinal tract, and pancreas (Akbas et al., 2009; Kendall and Yudowski, 2016). In contrast, the CB2R is located predominantly in the immune system (Akbas et al., 2009; Bermudez-Silva et al., 2010). Numerous studies have comprehensively demonstrated the efficacy of globally active first-generation CB1R antagonists in lowering body fat and ameliorating insulin resistance and dyslipidemia (Janero and Makriyannis, 2009; Le Foll et al., 2009; Chen et al., 2013; Lu et al., 2016). Therefore, CB1R has been regarded as an attractive therapeutic target for obesity for the past two decades (Di Marzo et al., 2011; Gatta-Cherifi and Cota, 2015; Kaur et al., 2016; Lu et al., 2016). The first-in-class selective CB1R antagonist and inverse agonist rimonabant (Acomplia; SR141716A), which was successfully approved and introduced into the European market in June of 2006, was anticipated to provide an effective treatment for obesity (Di Marzo, 2008; Sharma et al., 2014). Unfortunately, undesirable neuropsychiatric adverse effects, including anxiety, depression, and suicide were observed in some patients, and these central nervous system effects led to the withdrawal of SR141716A from the market in October of 2008 (Janero and Makriyannis, 2009; Le Foll et al., 2009).

The anti-obesity effects of CB1R antagonists could be attributed to a reduction in food intake (anorexia) due to the blockade of hypothalamic CB1Rs coupled with an increase in energy expenditure caused by peripheral CB1R blockade (Chorvat et al., 2012; Kaur et al., 2016; Lu et al., 2016). However, the persistent effect of SR141716A on body weight is mainly attributable to peripheral CB1R blockade because the anorexia was found to be transient, with tolerance developing within 1-2 weeks of the initial administration (Chen et al., 2010, 2011). Peripherally active CB1R inverse agonists or neutral antagonists, which are expected to show effective anti-obesity activities while circumventing the adverse central nervous system-associated effects of the first-generation brain-penetrating CB1R antagonists are, therefore, being investigated in several locations (Chorvat, 2013; Fulp et al., 2016; Knani et al., 2016; Lu et al., 2016; Iyer et al., 2017). Several excellent lead compounds such as TM-38837 with a brain to plasma distribution ratio (Kp) of 1/33 and JD-5037 (Kp < 1) have been reported recently. A second-generation CB1R antagonist (TM-38837) produced by 7TM Pharma has entered clinical trials for the treatment of obesity and metabolic disorders (Chorvat et al., 2012; Klumpers et al., 2013; Knani et al., 2016; Lu et al., 2016).

We have designed and synthesized a series of novel structural CB1R antagonists (4-methyl-1-hydrogen-diarylpyrazoles with modification at the pyrazole C-3 position) with the aim of restricting brain exposure while maintaining affinity and selectivity for peripheral CB1Rs. These compounds were based on our previous successful identification of several first-generation CB1R-selective antagonist candidates such as compounds MJ15 and MJ08, which could penetrate the blood–brain barrier (BBB) (Chen et al., 2010, 2011). Among the newly synthesized compounds, TXX-522 showed a minor brain presence while retaining high affinity and selectivity for the CB1R. In the present study, the pharmacological effects of TXX-522 on serum lipids, fat mass, glucose tolerance, and insulin sensitivity were explored in mice with high-fat diet-induced obesity (DIO).

## Materials and Methods

### Drugs and Chemicals

**FIGURE 1:**
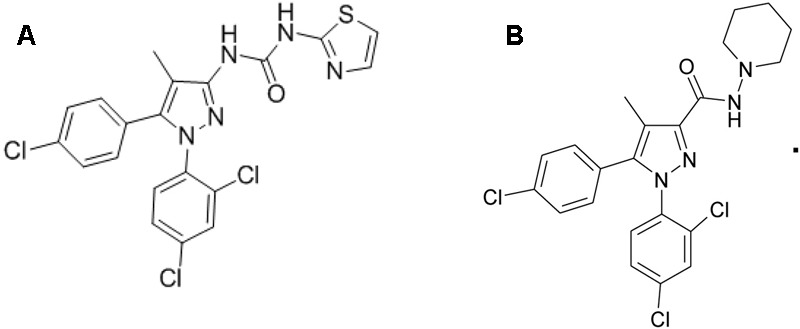
Chemical structures of **(A)** TXX-522 and **(B)** SR141716A.

Forskolin, WIN 55,212-2 and CP 55,940 (both CB1R/CB2R agonist), and fluoro-3 acetoxymethyl ester were purchased from Sigma–Aldrich Corp., (St. Louis, MO, United States). TXX-522 and SR141716A were synthesized and prepared by the New Drug Design Center of our Institute. Their purity and structure were confirmed using high-performance liquid chromatography, mass spectrometry, and proton (^1^H)-nuclear magnetic resonance. The structures of TXX-522 [(5-(4-chlorophenyl)-1-(2, 4-dichlorophenyl)-4-methyl-1H-pyrazol-3-yl) (piperidin-1-yl) methanone] and SR141716A are shown in **Figure 1**.

### CB1R Binding Mode Assay

The three-dimensional structure of CB1R was downloaded from the Protein Data Bank (code: 5TGZ) and prepared using Schrodinger 10.1 (Sastry et al., 2013). The three-dimensional structures of TXX-522 and SR141716A were built and optimized using Schrodinger 10.1. The two ligands were then docked individually into the refined CB1R using docking procedures within Schrodinger 10.1 (Friesner et al., 2006), and the docking pocket was centered on the ligand within the crystal structure of CB1R. No constraints were included while the Extra Precision docking mode was used (Alogheli et al., 2017).

### Competitive Radioligand Binding Assay

The affinities of TXX-522 for the human CB1R and CB2R receptor were analyzed using Cerep cooperation (Celle l’Evescault, France) using stably transfected CHO cells (CHO-hCB1R and CHO-hCB2R, respectively) and a previously reported competitive binding assay (Munro et al., 1993; Chen et al., 2011). Non-specific binding was determined in the presence of 10 μmol/L non-radioactive WIN 55,212-2. The affinity of TXX-522 for the CB1R or CB2R was expressed as an inhibitory constant (*K*_i_) value, based on the inhibition of the specific binding of radioactive [^3^H]-CP 55,940 to the relevant receptor. Each competitive binding inhibition curve was generated using data pooled from three independent experiments each conducted in duplicate.

### Cellular Assay of Cannabinoid Receptor Antagonism

Commercial enhanced green fluorescent protein (EGFP)-CB1/CB2_U2OS cells specifically designed to screen CB1R/CB2R agonists or antagonists (Thermo Fisher Bioimage ApS, Soeborg, Denmark) were used to examine the antagonist activity and selectivity of TXX-522 for these CBR subtypes. SR141716A was used as a reference compound in this assay. Based on a previously described method (Chen et al., 2011), the regular EGFP-CB1/CB2_U2OS cell culture medium was replaced with F12 culture medium containing 1 μmol/L Hoechst 33342 (Invitrogen, OR, United States) before each experiment, and incubated for 20 min at 37°C. Different concentrations (10 nmol/L, 30 nmol/L, 100 nmol/L, 300 nmol/L, 1 μmol/L, 3 μmol/L, and 10 μmol/L) of SR141716A or TXX-522 combined with WIN 55,212-2 (1 mmol/L), were then added. Dimethyl sulfoxide (DMSO, 0.1%) was used as the vehicle control. After further incubation for 2 h at 37°C, images of the cells were acquired using the IN Cell Analyzer 2000 (GE Healthcare, NJ, United States; 20× objective) and analyzed using the Granularity Analysis Module of the IN Cell. The inhibition ratio of each tested compound was expressed as percentage inhibition of the internalization induced by the reference CB1R/CB2R agonist, WIN 55,212-2. The data were used to calculate the half-maximal (50%) inhibitory concentration (IC_50_). The results were obtained from three independent experiments each performed in triplicate.

### Hippocampal Neurons and Fluoro-3 Acetoxymethyl Ester Ca^2+^ Imaging

Hippocampal neurons were prepared from neonatal rats as described previously and intracellular Ca^2+^ levels ([Ca^2+^]_i_) were investigated (Chen et al., 2010). Briefly, the neurons were loaded with a fluorescent Ca^2+^ indicator, fluoro-3 acetoxymethyl ester, and then treated with either 0.1% DMSO (vehicle control) or different concentrations of SR141716A or TXX-522 (10 nmol/L, 100 nmol/L, 1 μmol/L, or 10 μmol/L). WIN 55,212-2 (1 μmol/L) was added 1 min later. The fluorescence was monitored and imaged using the IN Cell Analyzer 1000 and analyzed using the Object Intensity Analysis Module of IN Cell. The [Ca^2+^]_i_ time courses were monitored by recording the relative fluorescence intensity per cell vs. time.

### Intracellular cAMP Assays

The functional antagonism of the response to a CB1R agonist was previously detected in the CHO-hCB1R and CHO-hCB2R cell lines (Chen et al., 2010, 2011). Intracellular cAMP was analyzed in these cells using the LANCE cAMP 384 kit (AD0262, PerkinElmer, San Jose, CA, United States) following exposure to the vehicle or different concentrations of the test compounds (individually or combined) in the presence of a phosphodiesterase inhibitor (2.5 μmol/L 3-isobutyl-1-methylxanthine) for 30 min. The cAMP level detected in the vehicle-treated cells was set as 1, and the levels detected in the treated cells were expressed as fold changes compared to the control level.

### *In Vivo* Studies

#### Animals

Male Sprague-Dawley rats (190–210 g) and 8-week-old Kunming or C57BL/6J mice were obtained from Vital River Laboratory Animal Technology Co., Ltd., (Beijing, China). The experimental protocols were performed strictly in accordance with the Guide for the Care and Use of Laboratory Animals of the National Institutes of Health (NIH) and were approved by the Institutional Animal Care and Use Committee of the Beijing Institute of Pharmacology and Toxicology.

#### Assay of Brain to Plasma Distribution of TXX-522

The compounds (2.5 mg/kg) were dissolved in 5% glucose solution containing polyoxyethylene castor oil (2.17%), 1,2-propanediol (0.56%), ethanol (0.75%), and medium chain triglycerides (0.48%) prior to intravenous administration to the Sprague-Dawley rats (*n* = 3), with an initial push of 0.3 mL at a speed of 75 mL/h, followed by 2.5 mL/h for 40 min. Retro-orbital blood samples (∼250 μL) were collected at defined time-points (0, 10, 15, 20, 25, 30, 35, and 40 min after dosing). The rats were then euthanized by decapitation, the brain and plasma samples were collected, and then stored at -80°C for subsequent assay. The plasma samples and whole rat brain, which had been homogenized with five volumes (v/w) of physiological saline on ice, were extracted by protein precipitation and analyzed using liquid chromatography-mass spectrometry/mass spectrometry, as previously reported (Fulp et al., 2012). The steady state Kp-value for each test compound was calculated and used as an index of brain penetration.

#### Assay of Effects of TXX-522 on WIN 55,212-2-Induced Hypothermia in C57BL/6 Mice

After a 7-day acclimation, body weight-matched male C57BL/6J mice (8-week-old) were randomly sorted into four different groups (*n* = 5 mice/group). Then, SR141716A (10 mg/kg) or TXX-522 (20 or 60 mg/kg) was orally administered 1 h prior to intraperitoneal administration of WIN 55,212-2 (3 mg/kg). The rectal temperature was monitored using a rectal probe coupled to a digital thermometer before and 30 min after treatment with WIN 55,212-2.

#### Assay of Effects of TXX-522 on Appetite in C57BL/6 Mice

Mice (8-week-old) were maintained singly for 7 days with *ad libitum* access to a standard diet before testing. On the test day, 20-h fasted mice were orally gavaged with the vehicle (4% DMSO and 4% Tween 20), SR141716A (10 mg/kg), or TXX-522 (20 or 60 mg/kg, *n* = 5 mice/group) 30 min before the onset of the dark cycle. At 30 min post treatment, the mice were allowed access to rodent chow, and their food intake was measured for the subsequent 3 h.

#### *In Vivo* Study of Effects of TXX-522 on Obesity in DIO C57BL/6 Mice

Male C57BL/6J mice (6-week-old) were maintained on a 12-h light/dark cycle at a controlled temperature (22 ± 1°C) and were fed a high-fat diet (45% fat, 18% protein, and 37% carbohydrate) for 20 weeks to establish the DIO mouse model (Chen et al., 2010, 2011). Age-matched standard diet-fed lean mice were used as the normal control group. The DIO mice were divided into the indicated groups (*n* = 8–9 mice/group) based on their initial body weights, and treated with SR141716A (5 mg⋅kg^-1^⋅day^-1^), TXX-522 (5 or 10 mg⋅kg^-1^⋅day^-1^), or the vehicle (1% DMSO) by gavage 1 h before the onset of the dark cycle daily for 4 weeks. Individual body weights and cage food consumption were measured daily. Oral glucose tolerance testing was performed on treatment day 23 on overnight-fasted mice while the serum glucose and insulin levels were assayed as described previously (Chen et al., 2013). At the end of the treatment period, the mice were fasted overnight prior to collecting blood samples for the analysis of serum triglycerides and total cholesterol levels as described previously (Xie et al., 2015). Furthermore, the intra-abdominal white adipose tissues (epididymal, lumbar, and perirenal) were removed and weighed.

### Statistical Analysis

All results are expressed as the mean ± standard error of the mean (SEM). For multiple comparisons, the statistical analysis was performed using a one-way analysis of variance, followed by Tukey’s multiple comparison test, using the statistical package for the social sciences (SPSS) version 11.5 (SPSS Inc., Chicago, IL, United States). A *P* < 0.05 was considered statistically significant.

## Results

### Binding Mode of TXX-522 with CB1R

**FIGURE 2:**
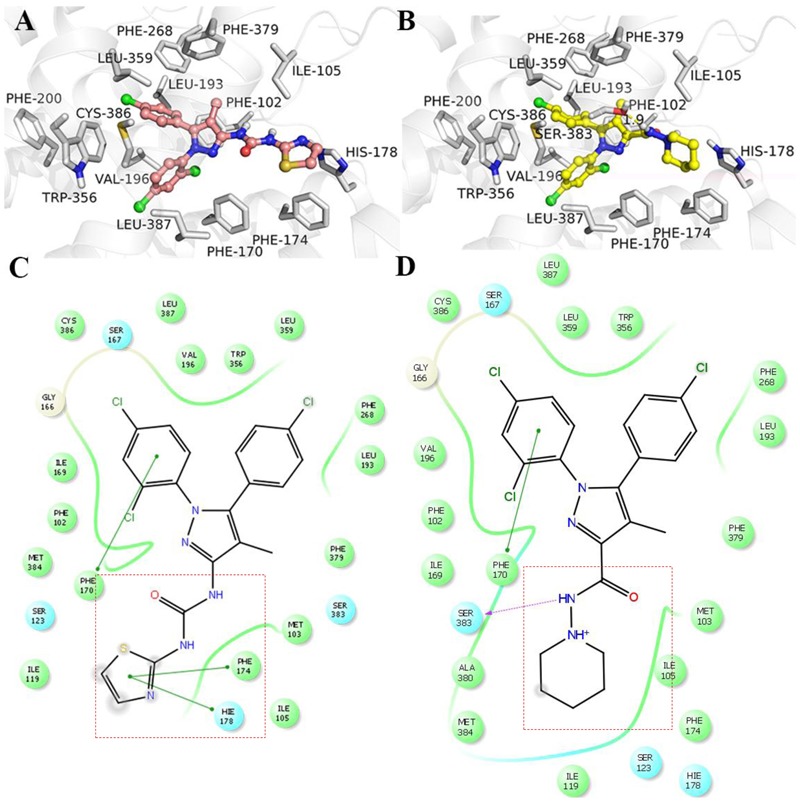
Interaction with cannabinoid receptor 1 (CB1R). **(A)** TXX-522 and **(B)** SR141716A docking with the CB1R. Two-dimensional topological representation of **(C)** TXX-522 and **(D)** SR141716A docking with CB1R.

The comparison of TXX-522 and SR141716A docking with the CB1R using Schrodinger 10.1 indicated that the two ligands had similar binding modes (**Figure 2**). The phenyl groups of TXX-522 and SR141716A were located in the hydrophobic pocket formed by the following residues: Phe102, Gly166, Ile169, Phe170, Leu193, Val196, Phe268, Trp356, Leu359, Phe379, Met384, Cys386, and Leu387. The ligands formed π–π stacking and hydrophobic interactions with these residues (**Figures 2A,B**). These hydrophobic interactions contributed to the potent affinities of TXX-522 and SR141716A for the CB1R (Hua et al., 2016; Shao et al., 2016). It is worth noting that a hydrogen bond interaction existed between the side chain of Ser383 and the amide group of SR141716A. However, no hydrogen bonds were found at the same position for TXX-522 because of the amide group was flipped by the introduction of the thiazole ring. Therefore, this analysis indicated that TXX-522 binds with the CB1R in a manner similar to SR141716A, but may have a weaker agonist activity than that of SR141716A because of the absence of hydrogen bonding.

### Selective Molecular and Cellular Interaction of TXX-522 with CB1R

**FIGURE 3:**
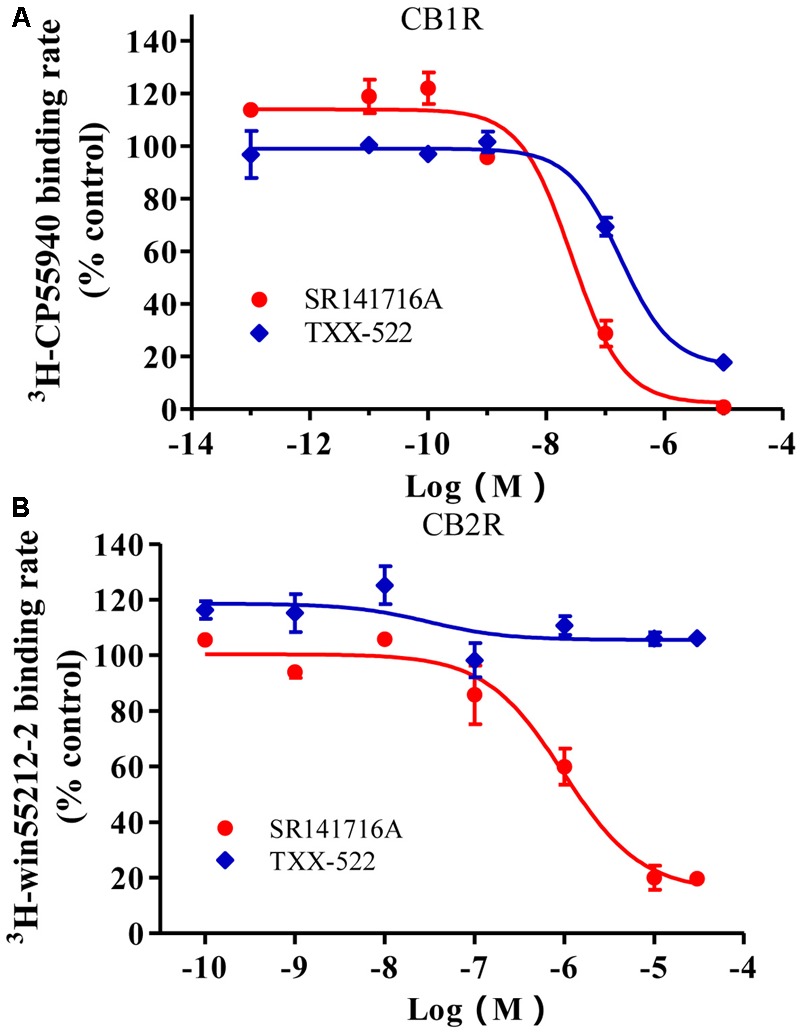
Competitive inhibition of radioligand binding to cannabinoid receptor 1 (CB1R) **(A)** and CB2R **(B)** in stably transfected CHO cells. Specific binding rates of 2.4 nmol/L [^3^H]-SR141716A to CHO cells expressing the CB1R and 2.4 nmol/L [^3^H]-WIN 55,212-2 to CHO cells expressing the CB2R were defined as 100%; [^3^H]-CP 55940 and [^3^H]-WIN 55,212-2 binding in the presence of indicated concentrations of SR141716A or TXX-522 were expressed relative to these values. Data are means ± SEM.

The CB1R inhibition and selectivity for the CB1R over the CB2R of the test compounds were first evaluated using a radioligand competitive binding assay, and then receptor redistribution analysis using EGFP-CB1_U2OS and EGFP-CB2_U2OS cell lines. The competitive binding inhibition curves for TXX-522 and SR141716A were similar in slope and shape (**Figure 3**). They both exhibited a sharply competitive concentration-dependent effect from 10^-9^ to 10^-6^ mol/L in the current assay. The concentration-response curves showed *K*_i_ and IC_50_ values of 0.15 and 0.17 μmol/L, respectively, for TXX-522 and the corresponding values for SR141716A were 0.015 and 0.013 μmol/L, respectively. In contrast, they both displayed much lower affinities for the CB2 receptor, with IC_50_ values > 30 and 1.6 μmol/L for TXX-522 and SR141716A, respectively. Therefore, the selectivity ratios were 176 and 106 for TXX-522 and SR141716A, respectively. However, the CB1/CB2 receptor agonist, CP 55,940, antagonized this effect, producing > 90% inhibition of the CB2 receptor at 100 nmol/L (data not shown).

Cannabinoid receptors are redistributed in cells after binding with an agonist and are activated following internalization ^[19]^. Therefore, internalization assays were performed on U2OS cells stably expressing EGFP-tagged human CB1R or CB2R (Chen et al., 2011) and exposed to TXX-522. Similar to SR141716A, TXX-522 concentration-dependently antagonized the internalization of the CB1-EGFP fusion protein induced by the CB1/CB2 receptor agonist, WIN 55,212-2 (1 μmol/L). Using the concentration-response curve, the IC_50_ value of TXX-522 for the CB1R was calculated as 10.33 ± 6.08 nmol/L, which was slightly lower than that of SR141716A (2.21 ± 0.21 nmol/L). In contrast, these compounds showed very weak antagonism of the CB2R, with CB1/CB2 receptor selectivity of 968 and 1400 for TXX-522 and SR141716A, respectively (**Table 1**). Taken together, these results indicate that TXX-522 was a selective CB1R agonist that effectively antagonized cellular CB1R activation.

**Table 1:** The antagonism of TXX-522 on the activation of the human CB1R or CB2R in EGFP-CB1_U2OS and EGFP-CB2_U2OS cells, respectively.

Compounds	M.W.	logP	tPSA	IC_50_(nmol/L) CB1R	IC_50_(μmol/L) CB2R	CB2R/CB1R
SR141716A	463.79	6.28	47.94	2.21 ± 0.12	3.23 ± 0.48	1461
TXX-522	547.96	7.95	56.73	10.33 ± 6.08	>10	>968

*EGFP-CB1_U2OS and EGFP-CB2_U2OS cells, specifically expressing CB1R and CB2R, respectively, were treated with vehicle or different concentrations of compounds, and then thee antagonistic activity of compounds to the CB1R or CB2R were detected by monitoring the internalization of the membrane localized CB1-, CB2-EGFP fusion protein to endosomes with the IN Cell Analyzer. CB1R, CB1 receptor; CB2R, CB2 receptor; tPSA, topological polar surface. The results were obtained from three independent experiments performed in triplicate. Values are means ± SEM.*

### BBB Permeability of TXX-522

The steady state distribution of TXX-522 in the brain and plasma was studied in normal rats, and the Kp was calculated as an index of brain penetration. Previous reports have indicated that compounds with a Kp-value of ≤0.1 are considered peripherally selective (Fulp et al., 2012, 2016). In the current study, the steady state concentrations of TXX-522 in brain and plasma were 528.9 ± 1.6 ng/mL and 23323.0 ± 267.8 ng/mL, respectively. Accordingly, the Kp-value of TXX-522 was only 0.02. In contrast, under the same conditions, SR141716A showed higher BBB permeability, with a Kp-value of 1.67. Thus, TXX-522 can be regarded as a promising candidate that mainly targets the peripheral tissues.

**FIGURE 4:**
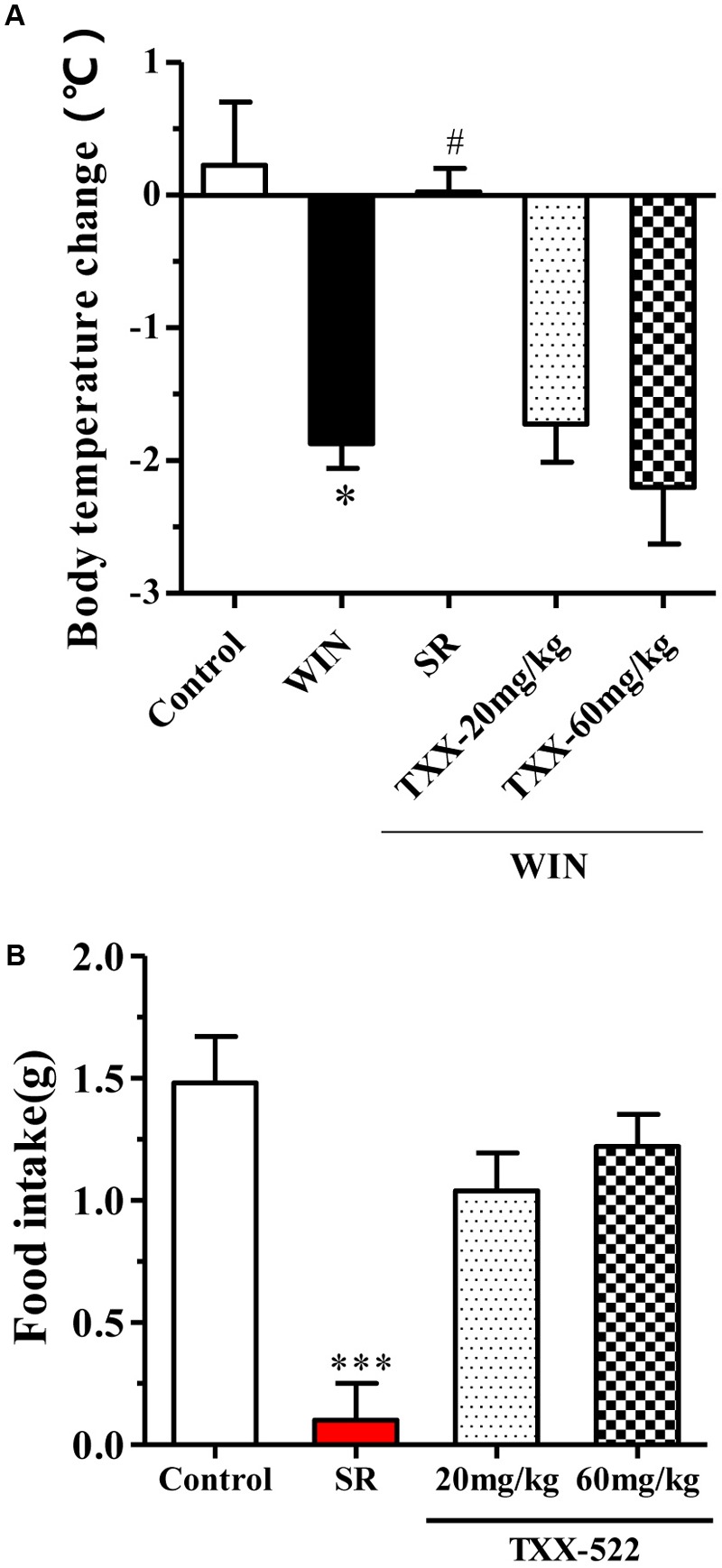
Effects of TXX-522 and SR141716A on WIN 55,212-2-induced hypothermia and acute food intake in C57BL/6 mice. **(A)** Body temperature change 30 min after intraperitoneal injection of WIN 55,212-2 (WIN, 3 mg/kg), following oral administration of SR141716A (SR, 10 mg/kg) or TXX-522 (TXX, 20 or 60 mg/kg). Data are means ± SEM, *n* = 5; ^∗^*P* < 0.05 vs. vehicle control group, ^#^*P* < 0.05 vs. the WIN 55,212-2-treated control mice. **(B)** Acute food intake for first 3 h of dark phase, which commenced 30 min after oral administration of SR141716A (10 mg/kg) or TXX-522 (20 or 60 mg/kg) to 24-h fasted mice via gavage. Data indicate the mean ± SEM, *n* = 5; ^∗∗∗^*P* < 0.001 vs. vehicle control group.

Previous reports have indicated that the central cannabinoid system is implicated in appetite regulation in rodents, where CB1R agonists can induce a tetrad of responses: hypothermia, hypoactivity, antinociception, and catalepsy (Kendall and Yudowski, 2016). To further examine the brain permeability of TXX-522 with respect to functional receptor activation, the effect of TXX-522 alone on core temperature and appetite, as well its effect on CB1R agonist-induced hypothermia were investigated *in vivo* in normal C57 mice. As expected, treatment with WIN 55,212-2 notably decreased the body temperature by acting on CB1R in the hypothalamus, and this effect was reversed by cotreatment with the brain-penetrating CB1R antagonist, SR141716A 10 mg/kg (**Figure 4A**). However, consistent with its low Kp-value, TXX-522 did not block WIN 55,212-2-induced hypothermia (**Figure 4A**), providing further evidence of its poor BBB permeability. Moreover, administration of TXX-522 alone did not affect the body temperature, suggesting a lack of CB1R inverse agonism (data not shown). In addition, the poor BBB permeability of TXX-522 was also reflected in the food intake assay, where this compound (20 and 60 mg/kg) did not affect acute food consumption by C57BL/6 mice; SR141716A (10 mg/kg) significantly inhibited food intake in this assay (**Figure 4B**).

### Effects of CB1R Antagonism of TXX-522 on Intracellular cAMP and [Ca^2+^]_i_

**FIGURE 5:**
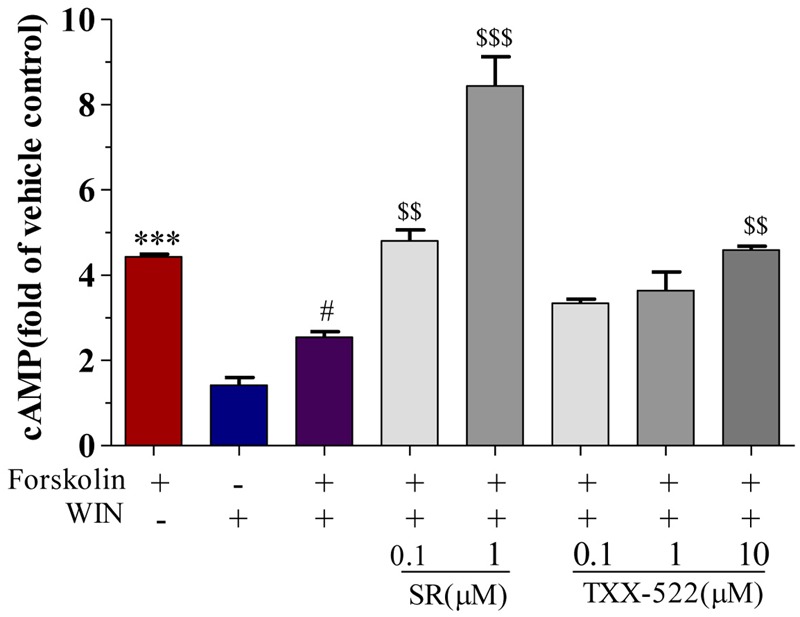
Effect of TXX-522 on WIN 55,212-2-mediated inhibition of forskolin-induced cAMP accumulation in CHO cells expressing human cannabinoid receptor 1 (CB1R). Data are expressed relative to cAMP level in control cells. Cells were exposed to 0.1 μmol/L forskolin. WIN, WIN 55,212-2 (1 μmol/L); SR, SR141716A. Data are means ± SEM, ^∗∗∗^*P* < 0.001 vs. vehicle control cells; ^#^*P* < 0.05 vs. forskolin-treated cells; ^$$^*P* < 0.01, ^$$$^*P* < 0.001 vs. forskolin plus WIN-treated cells.

**FIGURE 6:**
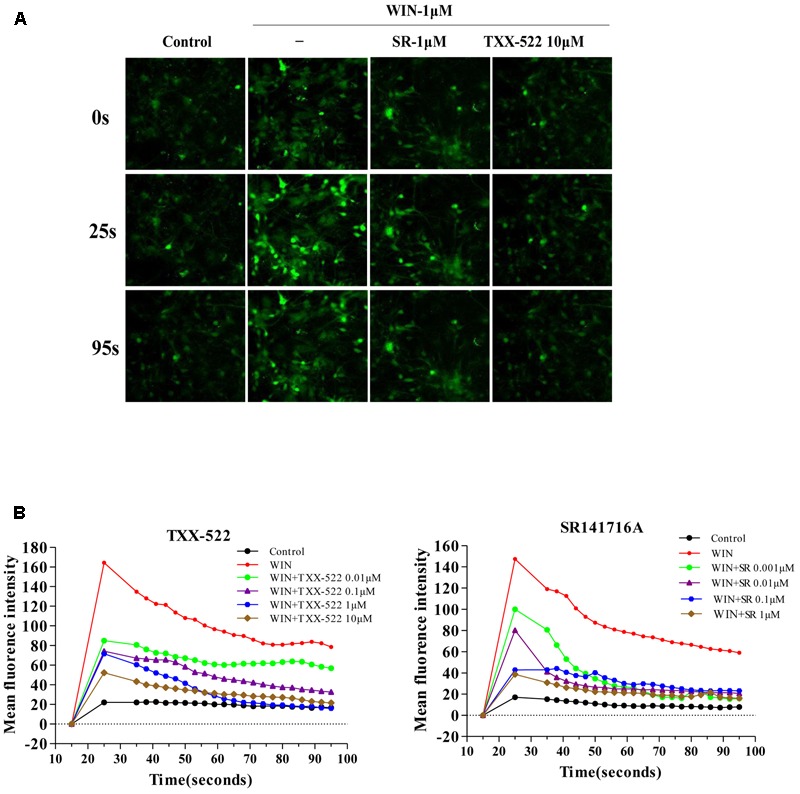
Effects of TXX-522 on WIN 55,212-2-mediated elevation of [Ca^2+^]_i_. **(A)** Representative micrographs showing [Ca^2+^]_i_ in hippocampal cells at indicated time-points. WIN, WIN 55,212-2; SR, SR141716A. **(B)** Dynamic course [Ca^2+^]_i_ elevation and pulse oscillation in cells exposed to indicated concentrations of WIN and TXX-522 or SR141716A.

cAMP and [Ca^2+^]_i_ are second messenger molecules in the CB1R signaling pathway. Activation of CB1R inhibits adenylate cyclase activity, rapidly reducing cAMP levels while [Ca^2+^]_i_ is increased by the modulation of voltage-gated calcium channels (N-type and P/Q-type) (Howlett, 2004; Mackie, 2006; Heifets and Castillo, 2009). To further investigate the specific effects of TXX-522 on CB1R function, we examined its effect on downstream signal transmission. TXX-522 dose-dependently reversed WIN 55,212-2-induced inhibition of forskolin-stimulated cAMP accumulation in CHO-hCB1 cells (**Figure 5**). The maximum effect observed at 10 μmol/L was equivalent to that induced by forskolin alone but was substantially lower than that induced by the same dose of SR141716A. Moreover, no further increase in the forskolin-induced cAMP level was observed with TXX-522 alone (up to 10 μmol/L, data not shown). This suggests that TXX-522 does not possess inverse agonist activity. Additionally, as expected, neither TXX-522 nor SR141716A affected the intracellular cAMP level in CHO-hCB2 cells, even at 10 μmol/L. Consistent with the cAMP result, our study in cultured rat hippocampal neurons showed that TXX-522 (10 nmol/L to 10 μmol/L) blocked the elevation and oscillation of [Ca^2+^]_i_ elicited by WIN 55,212-2 (1 μmol/L) in a concentration-dependent manner (**Figure 6**). Collectively, these results demonstrate that TXX-522 is a potent and selective CB1R antagonist that effectively antagonized intracellular signal transmission induced by CB1R activation.

### *In Vivo* Activity of TXX-522 in DIO Mice

**FIGURE 7:**
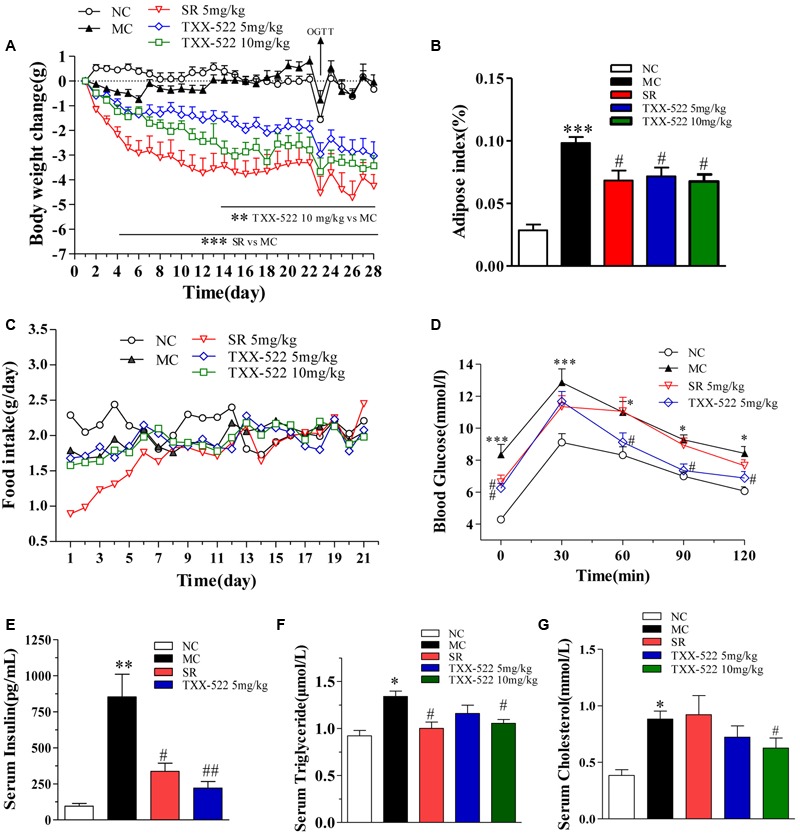
*In vivo* pharmacodynamic effects of TXX-522 and SR141716A in diet-induced obese (DIO) mice. **(A)** Body weight change was calculated individually and then averaged. **(B)** Intra-abdominal white adipose weight. **(C)** Food intake was calculated daily and then averaged. **(D)** Oral glucose tolerance testing was performed on treatment day 23 in overnight-fasted mice. **(E)** Fasted serum insulin. **(F,G)** Serum triglyceride and total cholesterol levels. Compounds were administered to DIO mice by oral gavage for 28 days. NC, normal control; MC, model control; SR, SR141716A. Data are means ± SEM of 8–9 animals per group. ^∗^*P* < 0.05, ^∗∗^*P* < 0.01, ^∗∗∗^*P* < 0.001 vs. NC group; ^#^*P* < 0.05, ^##^*P* < 0.01 vs. MC group.

The anti-obesity effect of TXX-522 was examined in DIO C57/B6 mice with a significantly increased body weight compared with that of the normal control mice (34.22 ± 1.19 g vs. 25.13 ± 0.87 g, *P* < 0.01) at the beginning of the experiment. The oral bioavailability and brain exposure of TXX-522 were also simultaneously assessed indirectly. Sequential monitoring showed that TXX-522 dose-dependently decreased the body weights of the DIO mice immediately after administration. Furthermore, a statistically significant difference was observed from 2 weeks until the end of the experiment (**Figure 7A**). However, a significant reduction in the body weight of SR141716A-treated mice was observed from day 4 onward. **Figures 7A,B** show the comparative loss of body weight and intra-abdominal adipose tissue at the end of the study in animals administered the two test compounds. In contrast to the reduced food intake of the SR141716A treatment group during the 1st week of administration, no anorexia was found in the TXX-522-treated mice (**Figure 7C**). Additionally, both SR141716A and TXX-522 dramatically decreased the fasting blood glucose and insulin levels, but oral glucose tolerance testing showed that TXX-522 (5 mg/kg) ameliorated glucose intolerance more than the same dose of SR141716A did (**Figures 7D,E**). SR141716A notably reduced hypertriglyceridemia (*P* < 0.05); however, a higher dose of TXX-522 (10 mg/kg) lowered both serum triglyceride and total cholesterol levels (**Figures 7F,G**). Additionally, no adverse effects on physical appearance, behavior, reaction to treatment, or other apparent signs of toxicity including lethargy, mania, or alteration of excreta were observed during the experiment in the TXX-522 treatment groups.

## Discussion

Based on the structure-activity analyzes of SR141716A, to lower the BBB permeability, highly polar hydrophilic groups such as guanidine, quaternary amine alkali, and uramido groups were introduced at the C-3 position of the pyrazole ring of SR141716A. A series of novel 1-(5-(4-chlorophenyl)-1-(2,4-dichlorophenyl)-4-methyl-1H-pyrazol-3-yl)-ureas were designed and synthesized. The introduction of a secondary amino group at the C-3 position generated TXX-522, which was identified as a highly selective CB1R antagonist with minimal BBB permeability (Kp = 0.02), although its CB1R affinity and *in vitro* bioactivity were slightly inferior to those of SR141716A. Preliminary pharmacodynamic evaluation in the mouse DIO model revealed that TXX-522 had a comparable anti-obesity effect to that of SR141716A, and more prominent ameliorated insulin resistance.

The binding affinity and selectivity of TXX-522 for two CBR subtypes were first analyzed at the molecular level using a radioligand competitive binding assay with purified CB1R and CB2R proteins. TXX-522 competed with [^3^H]-SR141716A for CB1R in a concentration-dependent manner and did not compete with the CB1R/CB2R agonist CP 55,940 for CB2R. The affinity of TXX-522 (IC_50_ 0.17 μmol/L) for CB1R was inferior (approximately one-tenth) to that of SR141716A (IC_50_ 0.013 μmol/L). This is consistent with the binding mode assay, where TXX-522 was predicted to have a relatively lower affinity than that of SR141716A for CB1R because it lacked a hydrogen bond interaction with the side chain of a key residue (Ser383) in the CB1R. Following this *in vitro* molecular evaluation, we investigated the cellular effects of these compounds. This analysis showed that TXX-522 displayed a more potent (approximately one-fifth of SR141716A) functional antagonism of EGFP-CB1 fusion protein internalization to endosomes, which reflects receptor activation. As reported in our previous study, this cellular high-content assay has advantages over *in vitro* molecular binding assays because it is performed in living cells and provides more valuable information relating to the binding, activation, and cell toxicity of the test compounds simultaneously in one assay (Chen et al., 2011). Collectively, these molecular and cellular assays demonstrated that TXX-522 was a selective CB1R antagonist that bound to the CB1R and effectively antagonized its activation with only a slight effect on CB2R activation.

The CB1R is a typical Gαi/o protein-coupled receptor, and its blockade has functional consequences for intracellular cascades, including elevation of the intracellular cAMP level and attenuation of the [Ca^2+^]_i_ elevation induced by receptor activation by a CB1R agonist (Chorvat, 2013; Gatta-Cherifi and Cota, 2015). Thus, the effect of TXX-522 on downstream signaling following receptor activation was assayed to further corroborate the efficacy of TXX-522 as a selective CB1R antagonist. Consistent with the characteristics of a classical CB1R antagonist, TXX-522 reversed the effects of a CB1R agonist (WIN 55,212-2) on forskolin-mediated changes in cAMP and [Ca^2+^]_i_ in a concentration-dependent manner. In contrast to SR141716A, which also acted as an inverse agonist of CB1R, TXX-522 showed no inverse activation of this receptor; no further increase in forskolin-stimulated cAMP accumulation was observed in CHO-hCB1 cells. In addition, TXX-522 did not affect the intracellular cAMP level in CHO-hCB2 cells. Therefore, these cellular functional assays further confirmed that TXX-522 acted as a selective CB1R antagonist.

A compound can be considered peripherally selective if it shows less than 10% brain penetration. The distribution of TXX-522 in brain tissue and plasma was first studied following intravenous administration to normal rats after distribution had reached a steady state as described previously (Wu et al., 2011). This analysis identified only 2% of TXX-522 in the brain tissue, and most of this compound was retained in peripheral tissues while under the same condition, >60% of SR141716A was distributed in the brain tissue. Consistent with this distribution, TXX-522 showed no effect on the CB1R agonist-induced hypothermia, which is one of the central tetrad of responses to CB1R agonist. In addition, TXX-522 did not affect the acute food consumption of normal C57BL/6 mice or the appetite of the DIO mice. As previously demonstrated, the suppression of food intake is related to modulation of the CB1R in the hypothalamus and mesolimbic regions, which are involved in appetite control (Kirkham et al., 2002). Taken together, these data indicate that TXX-522 is a selective CB1R antagonist with poor BBB permeability. According to the structure-activity relationship study, the low brain permeability of TXX-522 may reflect its relatively high molecular weight (547.96) and low lipophilicity, as indicated by its relatively higher topological polar surface (56.73 for TXX-522 and 47.94 for SR141716A; **Table 1**).

**FIGURE 8:**
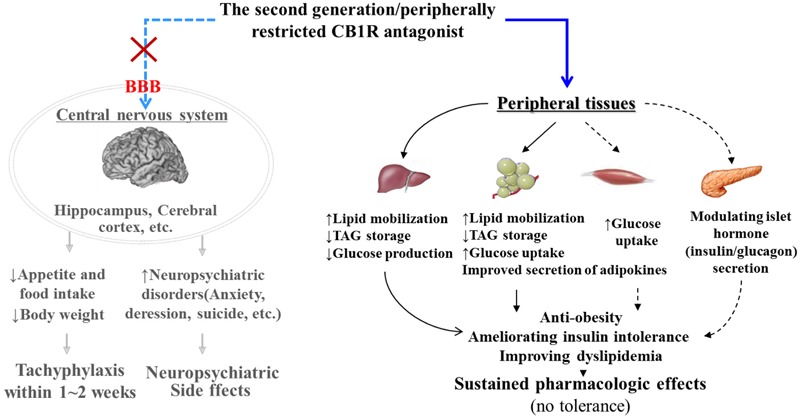
Pharmacological mechanism of peripherally acting cannabinoid receptor 1 (CB1R) antagonist. By specifically acting on the CB1R in peripheral tissues such as the liver, adipose tissue, muscle, and pancreatic β cells, peripherally restricted CB1R antagonists have the potential to combat obesity and related metabolic disorders without producing adverse central nervous system effects. Black bold line, direct roles; black dotted line, indirect roles or roles that require further confirmation. CB1R, cannabinoid 1 receptor; BBB, blood–brain-barrier; TAG, triglycerides.

Finally, we further evaluated the *in vivo* pharmacodynamic effects of TXX-522 in a commonly used mouse model of obesity. A preliminary screening test performed in DIO Kunming mice demonstrated that a 3-week treatment with 20 mg/kg TXX-522 significantly reduced body weight and fat mass (data not shown). Thus, DIO C57/B6 mice were treated with lower doses (5 and 10 mg/kg) of TXX-522 to compare its anti-obesity effect with that of SR141716A, as a reference compound. Consistently, chronic treatment with TXX-522 showed a comparable efficacy to that of SR141716A in inducing weight loss and decreasing abdominal fat in the DIO mice by the end of the study. However, SR141716A produced greater effects during the early period of the study, which may reflect the lack of TXX-522-induced anorexia. However, 5 mg/kg TXX-522 ameliorated the insulin resistance and hyperinsulinemia more than the same dose of SR141716A did. Thus, these *in vivo* pharmacological evaluations clearly demonstrated that TXX-522 was orally bioavailable and possesses CB1R-selective *in vivo* antagonist activity with minimal BBB permeability. Furthermore, this observation was confirmed by the unchanged food intake of TXX-522-treated DIO mice. These findings further corroborated the idea that compounds that interact with peripheral CB1Rs can produce many of the beneficial metabolic effects of globally active CB1R antagonists (**Figure 8**) (Chorvat et al., 2012; Lu et al., 2016). Certainly, these findings indicate that the further evaluation of the efficacy of TXX-522 in other models of obesity is warranted.

In summary, the current data demonstrate that TXX-522 is a potent and selective antagonist of the peripheral CB1R that deserves further development. This compound showed poor BBB penetration and lacked inverse agonist activity, which reduced its potential to cause adverse central effects such as those observed with rimonabant. In addition, TXX-522 has an advantage over several recently reported peripheral CB1R antagonists such as JD5037 and LH-21, which induced anorexia in DIO mice and rats (Alonso et al., 2012; Silvestri and Di Marzo, 2012).

## Author Contributions

Conceived and designed the experiments: WC and LW. Performed the experiments: FS, WC, and WL. Performed the binding mode assay: CL and WF. Analyzed the data: WC, FS, WF, and LW. Contributed reagents: XZ and ZZ. Wrote the paper: WC and LW.

## Conflict of Interest Statement

The authors declare that the research was conducted in the absence of any commercial or financial relationships that could be construed as a potential conflict of interest.
