# Corrigendum: Novel Peripherally Restricted Cannabinoid 1 Receptor Selective Antagonist TXX-522 with Prominent Weight-Loss Efficacy in Diet Induced Obese Mice

**DOI:** 10.3389/fphar.2017.00890

**Published:** 2017-11-28

**Authors:** Wei Chen, Fengchun Shui, Cheng Liu, Xinbo Zhou, Wei Li, Zhibing Zheng, Wei Fu, Lili Wang

**Affiliations:** ^1^Beijing Institute of Pharmacology and Toxicology, Beijing, China; ^2^State Key Laboratory of Toxicology and Medical Countermeasures, Beijing, China; ^3^Department of Medicinal Chemistry, School of Pharmacy, Fudan University, Shanghai, China

**Keywords:** CB1R antagonist, obesity, periphery, blood–brain-barrier

There is an error in the Funding statement. The correct number for **the Major Program of the Ministry of Science and Technology of China** is 2012ZX09301-001, 2012ZX09301-003.

In the original article, we neglected to include the funder **the National Natural Science Foundation of China**, **81430090** to **LW**.

The authors apologize for these errors and state that this does not change the scientific conclusions of the article in any way.

## Conflict of interest statement

The authors declare that the research was conducted in the absence of any commercial or financial relationships that could be construed as a potential conflict of interest.

